# *In Vitro* Antioxidant Activities, Free Radical Scavenging Capacity, and Tyrosinase Inhibitory of Flavonoid Compounds and Ferulic Acid from *Spiranthes sinensis* (Pers.) Ames 

**DOI:** 10.3390/molecules19044681

**Published:** 2014-04-15

**Authors:** Chung Pin Liang, Chia Hao Chang, Chien Cheng Liang, Kuei Yu Hung, Chang Wei Hsieh

**Affiliations:** 1 Department of Medicinal Botanicals and Health Applications, Da-Yeh University, Changhua 51591, Taiwan; E-Mail: q8528852@hotmail.com; 2 Cheng Ching General Hospital, Taichung 42881, Taiwan; E-Mail: hideben@hotmail.com; 3 Horien International Co., Ltd., Taichung 42881, Taiwan; E-Mail: jasonliang@hydron.com.tw; 4 Pemay Biomedical Technology Corp, Taichung 41348, Taiwan; E-Mail: pemay321@gmail.com

**Keywords:** *Spiranthes sinensis*, ultrasound-assisted extraction, ferulic acid, flavonoids, antioxidant activity, tyrosinase

## Abstract

In this study, ultrasound-assisted extraction (UAE) and other methods of extracting flavonoid compounds and ferulic acid (FA) from *S. sinensis* were investigated. Five different extraction methods, including water extraction (W), water extraction using UAE (W+U), 75% ethanol extraction (E), 75% ethanol extraction using UAE (E+U), and supercritical CO_2_ extraction (SFE) were applied in the extraction of bioactive compounds (flavonoids and ferulic acid) in order to compare their efficiency. The highest yield of flavonoids (4.28 mg/g) and ferulic acid (4.13 mg/g) content was detected in the E+U extract. Furthermore, *S. sinensis* extracts obtained by E+U show high antioxidant activity, and IC_50_ values of 0.47 mg/mL for DPPH radicals and 0.205 mg/mL for metal chelating activity. The total antioxidant assay shows superoxide radical scavenging capacity and *in vitro* mushroom tyrosinase inhibition in a dose-dependent manner, suggesting that E+U can be used for extraction of bioactive compounds from *S. sinensis*.

## 1. Introduction

*Spiranthes sinensis* (Pers.) Ames is a famous Traditional Chinese Medicine herb which is widely used in the treatment of bacterial and inflammatory diseases, cancer and blood and chest disorders [1,2]. Previous investigations of *S. sinensis* have yielded flavonoids, homocyclotriucallane, dihydro-phenanthrenes and ferulic acid (FA) [3,4].

Flavonoids comprise the major class of phenolic compounds widely present in natural plant tissues [5]. They are highly effective antioxidants and less toxic than synthetic antioxidants [6]. Isolated flavonoid compounds from *Trifolium nigrescens* Subsp. *Petrisavi* have antioxidant and tyrosinase inhibitory activities [7]. FA is a hydroxycinnamic acid widely present in plants and vegetable foods. Its biological properties such as antioxidant activity and tyrosinase inhition are well recognized [8,9]. The special structure of FA also endows it with strong UV-absorptive ability, making it an important skin-protecting agent [10]. All this justifies the great attention paid to finding an effective method to extract flavonoids and FA from *S. sinensis.*

At present, commonly used extraction methods include: solvent extraction, Soxhlet extraction and microwave-assisted extraction. These methods have both advantages and disadvantages. For example, solvent extraction can result in polarity differences in the compounds, and the extracted substance may remain toxic; Soxhlet extractions can reduce the amount of solvent needed, but require a long extraction time; and microwave-assisted extraction provides very rapid and efficient extraction [11], but the equipment is expensive. In order to seek more environmentally friendly methods which would decrease solvent consumption, shorten extraction times, increase extraction yields and enhance the quality of extracts, ultrasound-assisted extraction (UAE) and supercritical carbon dioxide extraction (SFE) have been developed. Compared with other extraction techniques, UAE and SFE are efficient alternatives, and their operations are easier [12,13]. UAE has proved to be a particularly effective extraction method to reduce the extraction temperature and amount of solvent and shorten the extraction time, which is especially useful for the extraction of thermosensitive and unstable compounds. Therefore, UAE has been widely used in the literature for the extraction of biologically active compounds, including the extraction of polyphenols and flavonoids from yellow tea [14]. In recent years, many studies have been done to use SFE with carbon dioxide (CO_2_) as a solvent for extraction of natural compounds from different raw materials [15]. The combined liquid-like solvating capabilities and gas-like transport properties of supercritical fluids make them particularly suitable for extracting bioactive compounds from plant tissues with a high degree of recovery in a short period of time. It was believed that, by using SFE, the extraction time can be reduced to tens of minutes compared with liquid–solid extraction that requires hours or days [16].

It has been reported that some extraction methods have been used for extracting bioactive compounds from *S. sinensis*. Zhao *et al.* [17] extracted flavonoid compounds from *S. sinensis* as follows: extraction time 3 h, 75% ethanol, and a solid-to-solvent ratio of 1:40. Those extractions are similar to ours, however, their extractions did not use UAE or controlled temperature and required a long extraction time. Dong *et al.* [18] and Peng *et al.* [2] used a large amount of *S. sinensis* powder: 10 and 5 kg, respectively, for reflux extraction to extract flavonoids. 

Therefore, the aim of this study was to investigate the most efficient way of extracting flavonoid compounds and FA from *S. sinensis* and to evaluate the antioxidant activities and *in vitro* tyrosinase activity in the extracts obtained by E+U.

## 2. Results and Discussion

### 2.1. Discussion of the Five Different Extraction Methods to Obtain a Higher Content of Flavonoids and Ferulic Acid

**Figure 1:**
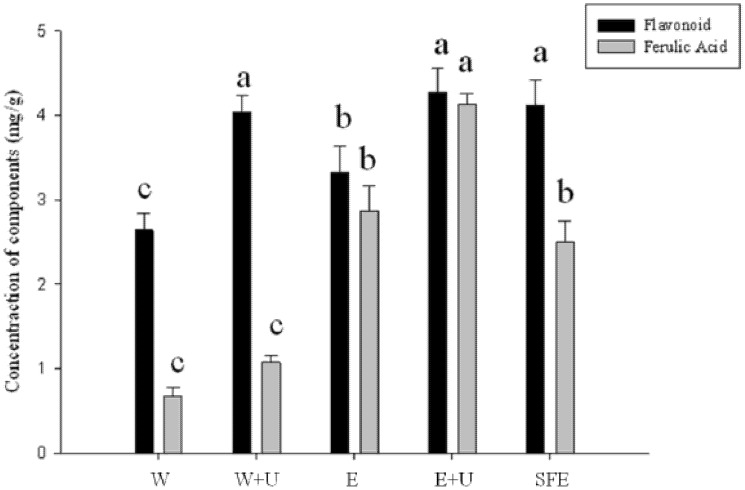
Comparison of the five extraction methods for obtaining flavonoids and FA. (1) W, water extraction; (2) W+U, water extraction using UAE; (3) E, 75% ethanol extraction; (4) E+U, 75% ethanol extraction using UAE; (5) SFE, supercritical CO_2_ extraction. a, b, c describe bioactive compound levels: a, the highest then the others; c, the lowest then the others; b, middle of a and c.

Figure 1 shows our results for the five different extraction methods: water extraction (W), water extraction using UAE (W+U), 75% ethanol extraction (E), 75% ethanol extraction using UAE (E+U) and supercritical CO_2 _extraction (SFE). The W extraction had a flavonoid content of 2.64 mg/g and FA content of 0.68 mg/g; W+U extraction had a flavonoid content of 4.04 mg/g and FA content of 1.08 mg/g; E extraction had a flavonoid content of 3.32 mg/g and FA content of 2.87 mg/g; E+U extraction had a flavonoid content of 4.28 mg/g and FA content of 4.13 mg/g; and SFE extraction had a flavonoid content of 4.12 mg/g and FA content of 2.50 mg/g. The total yields of the extractions by W, W+U, E, E+U and SFE extraction were thus 2.8%, 3.4%, 3.2%, 4% and 7.4%, respectively. The flavonoid and FA yield ratio of different extraction methods were as follows: in solvent extraction methods, the flavonoid and FA yield ratio of the E extraction (3.32 mg/g, 2.87 mg/g) were higher than that of the W extraction (2.64 mg/g, 0.68 mg/g). In the ultrasound extraction methods, the flavonoid and FA yield ratios of E+U extraction (4.28 mg/g, 4.13 mg/g) were higher than those of the W+U extraction (4.04 mg/g, 1.08 mg/g). These results indicated that the E+U extraction was more efficient in extracting flavonoids and FA from *S. sinensis*.

In the Lina *et al.* study [19], the ethanol extraction resulted in higher phenolic compounds than the water extraction from coffee silverskin. A comparison of the ultrasound and non-ultrasound extraction of flavonoids from yellow tea showed that the flavonoid content produced by ultrasound-assisted extraction at 75% ethanol was higher than the others (water, 75% ethanol, ultrasound-assisted extraction with water) [14]. This study proves that E+U extraction could successfully be used for extraction of flavonoids and FA from *S. sinensis*.

In our results, the SFE extraction of flavonoid compounds and FA from *S. sinensis* was less effective than that of ultrasonic extraction, possibly because the pressure (14 MPa) was not high enough. In Bruni *et al.*’s study [20], the use of ultrasonic extraction was compared with SFE for extracting vitamin E from *Amaranthus caudatus* seeds, and the results showed that the SFE extraction of vitamin E was superior to the UAE method. They used a pressure of 40 MPa, but the energy losses increased, with a resulting relative increase in the cost.

These studies have suggested that UAE is more efficient than conventional solvent extraction without UAE in extracting bioactive compounds from different natural sources. UAE was faster and more efficient in extracting bioactive components due to the strong disruption of sample tissue structure under ultrasonic acoustic cavitation. The E+U extraction was chosen for extracting the total flavonoid components and FA used for further antioxidant activities and *in vitro* tyrosinase activity.

### 2.2. DPPH Radical Scavenging Capacity

**Figure 2:**
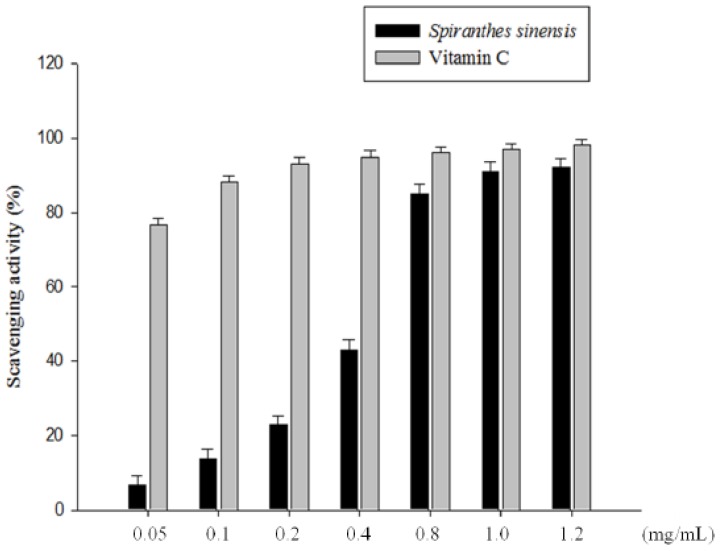
DPPH radical scavenging capacity of *S. sinensis* E+U extract. The DPPH scavenging effects of the extract at different concentrations compared with vitamin C. Results are represented as percentages of control, and the data are mean ± SD for the three separate experiments.

After determining E+U extraction to be the most efficient extraction process of *S. sinensis*, we investigated the antioxidant abilities of *S. sinensis* extract processed under specific extraction conditions, as the antioxidant bioactivities of plants might be a main contributor to their biological functions, including their hepetoprotective properties. The DPPH scavenging effects increased with increased concentrations of *S. sinensis* extract (Figure 2). The IC_50_ of DPPH radical scavenging activity of *S. sinensis* extract was 0.47 mg/mL; it showed 92% DPPH radical scavenging ability at a concentration of 1.2 mg/mL, similar to vitamin C. You *et al.*’s [21] results show that the ethanol extract and purified pigments from *Castanea mollissima* shells (CPS) possessed excellent DPPH scavenging capacity. Flavonoids have a diphenylpropane structure, in which the B or A ring of the structure contains catechol: C-3 includes either hydroxy or gallic acid (galloyl group), C-2 and C-3 are linked by a double bond and C-4 is in a keto form. These provide flavonoids with their free radical scavenging capacity [22]. 

In the antioxidant system, oxidation is accompanied by reduction. An antioxidant index is the measurement of the reducing power [23]. The antioxidant capacity can be measured from absorbance values; the higher the absorbance, the greater the antioxidant capacity. As indicated in Figure 2, the higher the concentration of vitamin C and *S. sinensis* E+U extract, the greater the antioxidant property.

Although the data (Figure 2) showed that *S. sinensis* E+U extract had a slightly lower free radical scavenging effect and reducing power than synthetic vitamin C, *S. sinensis* E+U extract is a natural product with an efficient scavenging power that should have a higher commercial value.

### 2.3. Metal Chelating Activity

**Figure 3:**
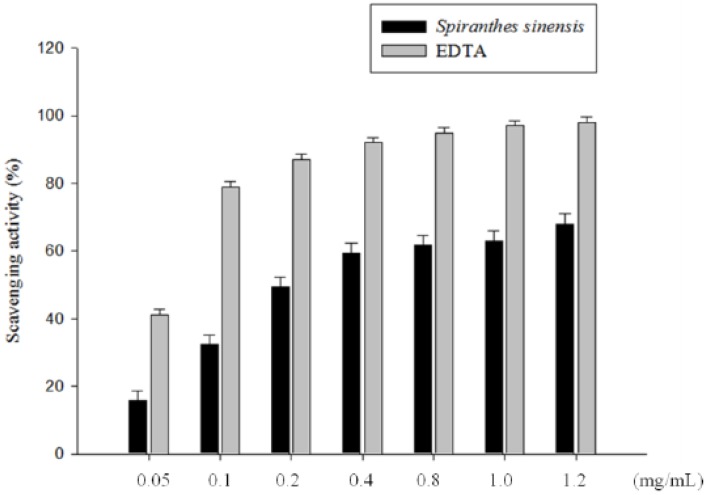
Metal-ion chelating activity of *S. sinensis* E+U extract. Various concentrations of the extract or EDTA were used in the study. Results are represented as percentages of control, and the data are mean ± SD for the three separate experiments.

Ferrozine can quantitatively form complexes with Fe^2+^. In the presence of other chelating agents or antioxidants, the complex formation is disrupted, with the result that the purple color of the complexes decreases. The effect of *S. sinensis* E+U extract on the chelating ability of ferrous ion is shown in Figure 3. As can be seen, the IC_50_ of the metal-ion chelating capacity of *S. sinensis* E+U extract (0.05, 0.1, 0.2, 0.4, 0.8, 1.0 and 1.2 mg/mL) was 0.205 mg/mL, for a metal-ion chelating ability of 92% at a concentration of 1.2 mg/mL. Furthermore, the metal chelating activity of the obtained extracts was slightly lower than that of EDTA at the same concentration. This result indicated that *S. sinensis* extract obtained by UAE had significant metal chelating activity; furthermore, the effect increased with increases in the concentration of *S. sinensis* extract.

### 2.4. Total Antioxidant Activity

**Figure 4:**
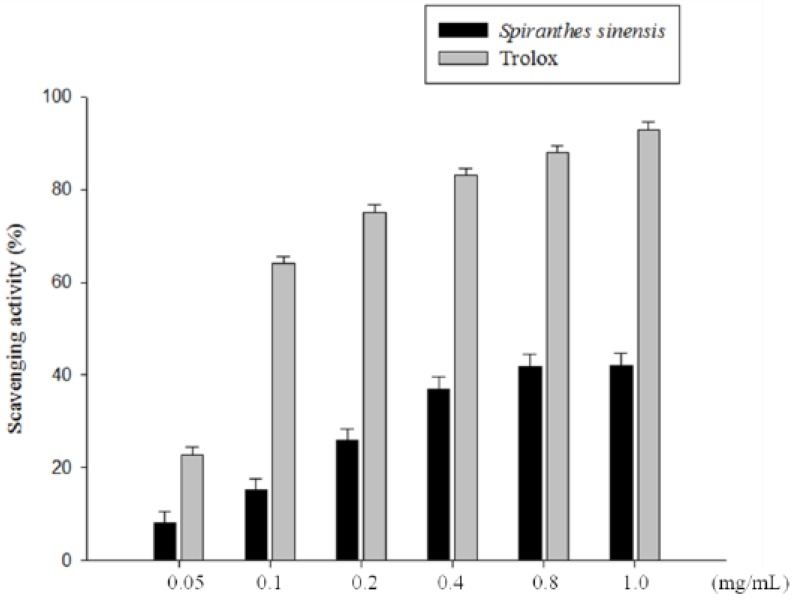
Total antioxidant activity of *S. sinensis* E+U extract. Different concentrations of the extract or Trolox were used in the study. Results are represented as percentages of control, and the data are mean ± SD for the three separate experiments.

Figure 4 shows the total antioxidant ability of *S. sinensis* E+U extract as compared with Trolox as the control standard. It appeared that the total antioxidant ability of the obtainedextracts was primarily related to their concentrations. The total antioxidant activity of the *S. sinensis* E+U extract (0.05, 0.1, 0.2, 0.4, 0.8 and 1.0 mg/mL) assay was compared with that of Trolox. At a concentration of 1.0 mg/mL, the *S. sinensis* E+U extract total antioxidant ability was 42.0%, and standard Trolox was 93%. In the present study, the *S. sinensis* E+U extract thus showed lower free radical scavenging activities as compared to Trolox.

It has been reported that bioactive compounds extracted by solvent extraction and partitioned by ethyl acetate show good DPPH radical and ABTS scavenging capacity [24]. However, in previous studies, the extraction methods used to extract bioactive compounds from *S. sinensis* were complex. In our study, the E+U extraction was simple and required a short extraction time to extract bioactive compounds from *S. sinensis*.

### 2.5. Assay of Superoxide Radical Scavenging Capacity

**Figure 5:**
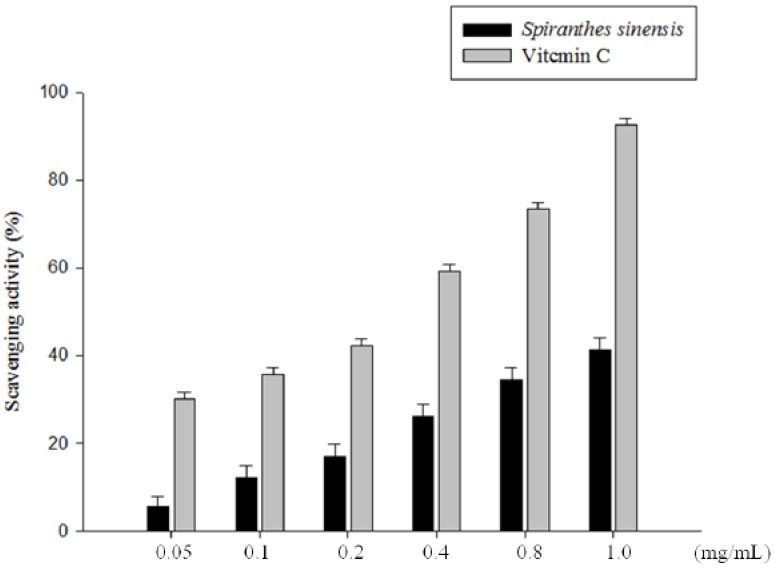
Superoxide radical scavenging capacity of *S. sinensis* E+U extract. The scavenging effects of the extract at different concentrations compared with vitamin C. Results are represented as percentages of control, and the data are mean ± SD for the three separate experiments.

Superoxide anions are generated by the oxidation of pyrogallol, and the scavenging effects are expressed as the inhibition of pyrogallol autoxidation, so any substance existing in the reaction system that might affect the oxidation of pyrogallol might also affect the test results. The *S. sinensis* E+U extract inhibited the hydroxylation of salicylic acid by reactive oxygen species in a dose-dependent manner. The reduction of total oxidation products as a function of the volume of the extract added to the assay is shown in Figure 5.

The superoxide radical scavenging activities of *S. sinensis* E+U extract and Vitamin C (0.05, 0.1, 0.2, 0.4, 0.8, 1.0 and 1.2 mg/mL) are shown in Figure 5. At a concentration of 1.0 mg/mL, the scavenging capacities for *S. sinensis* E+U extract and Vitamin C were 41.2% and 92.6%, respectively. Thermodynamically, the sample that has a redox potential lower than that of ABTS^+^ may react with the radical. Although superoxide anion is a weak oxidant, it gives rise to a generation of powerful and dangerous hydroxyl radicals, as well as singlet oxygen, both of which contribute to oxidative stress [25]. The results of Sakthidevi and Mohan [26] clearly indicated that *Dioscorea alata* leaf extracts have a noticeable effect as a scavenging superoxide radical because of their higher total phenolic and flavonoid contents.

### 2.6. Inhibitory Effect of S. sinensis E+U Extract on Mushroom Tyrosinase Activity

**Figure 6:**
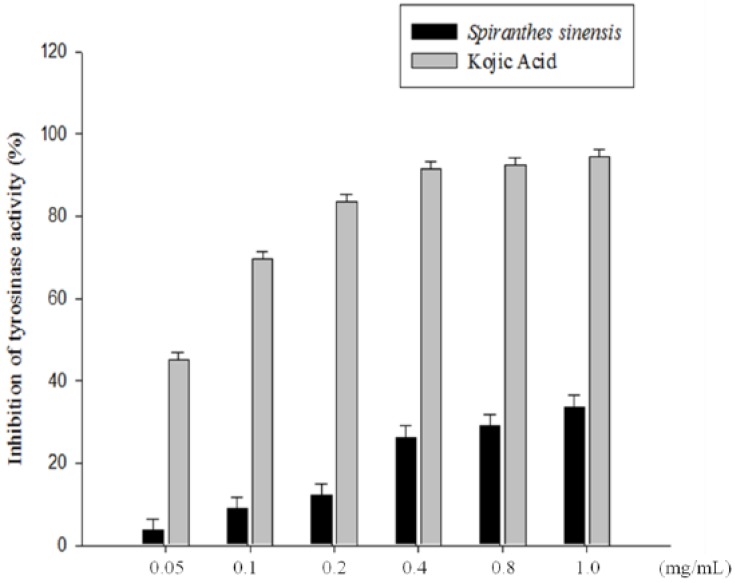
Inhibitory effect of *S. sinensis* E+U extract on mushroom tyrosinase activity. Different concentrations of the extract or kojic acid (0.05, 0.1, 0.2, 0.4, 0.8 and 1.0 mg/mL) were incubated with the same units of mushroom tyrosinase. Results are presented as mean ± SD for the three separate experiments

Tyrosinase plays an essential role in the melanin synthesis pathway, as it can convert l-tyrosine to l-DOPA and oxidize l-DOPA to form dopachrome. Mushroom tyrosinase is widely used as the target enzyme in screening potential inhibitors of melanogenesis. In order to analyze the *S. sinensis* E+U extract on mushroom tyrosinase activity, tyrosinase enzyme inhibition experiments were carried out in triplicate. The inhibitory effect of *S. sinensis* E+U extract on mushroom tyrosinase activity is shown in Figure 6. The results show that the extract reduced mushroom tyrosinase activity in a dose-dependent manner, although it had a slightly lower inhibitory effect on mushroom tyrosinase activity than kojic acid dose did. At a concentration of 1.0 mg/mL, the *S. sinensis* E+U extract inhibitory tyrosinase activity was 33.5%. Demirkiran *et al.* isolated 12 flavonoid compounds from *Trifolium nigrescens* Subsp. *Petrisavi* were evaluated for their antioxidant activity and inhibitory activity on mushroom tyrosinase [8]. Flavonoids, which are one of the most investigated groups of plant secondary metabolites, show tyrosinase inhibition [27]. Hence, *S. sinensis* E+U extract might act as a tyrosinase inhibitor. We also determined the effect of *S. sinensis* E+U extract on intracellular tyrosinase activity and melanin content in B16F10 cells. *S. sinensis* E+U extract significantly reduced the tyrosinase activity of α-MSH-stimulated B16F10 cells 15.87% at 60 μg/mL, the melanin production was suppressed 23.27% at 60 μg/mL (data not shown). In addition, the E+U extract of *S. sinensis* neither inhibited mushroom tyrosinase nor suppressed intracellular tyrosinse activity in B16F10 cells. 

## 3. Experimental

### 3.1. Plant Material

Whole plants of *S. sinensis* were purchased in April 2010 from a local herbal medicine store in Changhua City, Taiwan. After collection, the *S. sinensis* samples were stored at −20 °C. Before the extraction procedures, all frozen samples were freeze-dried, then crushed by the homogenizer, sieved through a 60-mesh screen and, finally, kept in the desiccator in properly sealed containers.

### 3.2. Chemicals

Aluminum chloride hexahydrate (AlCl_3_), potassium acetate (CH_3_COOK), quercetin, ferulic acid, 1,1'-diphenyl-2-2'-picrylhydrazyl (DPPH), ethylenediaminetetraacetic acid (EDTA), 2,2'-azino-bis(3-ethylbenzothiazoline-6-sulphonic acid (ABTS), 6-hydroxy-2,5,7,8-tetramethylchromane-2-carboxylic acid (Trolox), ferrozine, L-ascorbic acid (vitamin C), mushroom tyrosinase, l-3,4-dihydroxyphenylalanine (l-DOPA) and kojic acid were purchased from the Sigma Chemical Co. (St. Louis, MO, USA). All other chemicals used were of analytical grade.

### 3.3. Extraction Procedures

Five types of extraction methods were used in this study: water extraction (W), water extraction using UAE (W+U), 75% ethanol extraction (E), 75% ethanol extraction using UAE (E+U) and supercritical CO_2 _extraction (SFE):

Method 1 (W). Ten g of *S. sinensis* powder were added to 400 mL of water and then extracted at 60 °C for 30 min.
Method 2 (E). Ten of *S. sinensis* powder were added to 400 mL of 75% ethanol and then extracted at 60 °C for 30 min.
Method 3 (W+U). Ten g of *S. sinensis* powder were added to 400 mL of water, and then extracted with an ultrasonic frequency of 40 kHz at 60 °C for 30 min.
Method 4 (E+U). Ten g of *S. sinensis* powder were added to 400 mL of 75% ethanol, and then extracted with an ultrasonic frequency of 40 kHz at 60 °C for 30 min.
Method 5 (SFE). Ten g of *S. sinensis* powders and 400 mL of 75% ethanol were mixed; the mixture was then placed in the extraction tank for 30 min at 45 °C under 18 MPa to complete the extraction. The dynamic extraction mode was applied, and the flow rate of CO_2_ was 0.2 L/min. SFE was performed using a TS09-110 apparatus (Taiwan Supercritical Technology Co., Ltd., Changhua, Taiwan) by a procedure modified from the study of Chiu *et al.* [28].

After the *S. sinensis* extraction processes, all of the extracts were filtered by a 0.45 μm filter and kept at –20 °C for 24 h, after which the extracts were freeze-dried and store at 4 °C for experimental use. 

### 3.4. Determination of Total Flavonoid Compounds

The total flavonoid content was determined according to the aluminum chloride colorimetric E+U described by Chang *et al.* [29]. Briefly, aliquots of *S. sinensis* extract (0.1 g) were dissolved in deionized water (1 mL). A portion of this solution (0.5 mL) was mixed with 95% ethanol (1.5 mL), 10% AlCl_3_ (0.1 mL), 1 M CH_3_COOK (0.1 mL) and deionized water (2.8 mL). After incubation at room temperature for 40 min, the reaction mixture absorbance was measured at 415 nm against a de-ionized water blank on a spectrophotometer (Metertech SP8001, Taipei, Taiwan). Quercetin was chosen as a standard. Using a seven-point standard curve (0–50 mg/L), the levels of total flavonoid content in the *S. sinensis* extracts were determined in triplicate.

### 3.5. Determination of Ferulic Acid

The FA content of the five extracts was determined as per the Yang *et al.* method [30], with modifications. In brief, ferulic acid dissolved in 70% ethanol was the standard (5.0, 10.0, 15.0, 20.0, 25.0 μg/mL). Absorbance was then measured at 320 nm by a spectrophotometer. Then, the five *S. sinensis* extracts (1 mg) were dissolved with 70% ethanol (10 mL) and measured at 320 nm.

### 3.6. In Vitro Evaluation of Antioxidant Activities

#### 3.6.1. DPPH Radical Scavenging Capacity

The DPPH radical scavenging activities of *S. sinensis* E+U extract were determined by the method of Shimada [31], with minor modifications. Different concentrations of *S. sinensis* E+U extract were mixed with 0.1 mM DPPH dissolved in methanol (5 mL). An ethanol solution of the samples (1 mL) with various concentrations (0.05, 0.1, 0.2, 0.4, 0.8, 1.0 and 1.2 mg/mL) was mixed with 0.1 mM DPPH dissolved in methanol (5 mL). The mixture was incubated at room temperature in the dark for 20 min. The control contained all the reagents without the sample and was used as a blank. The DPPH radical scavenging capacity was determined by measuring the absorbance at 517 nm using a spectrophotometer. The DPPH radical scavenging capacity of vitamin C was also determined for comparison. The DPPH radical scavenging capacity was calculated as (1 − absorbance of sample/absorbance of control) × 100%.

#### 3.6.2. Metal Chelating Activity

The metal chelating activities of *S. sinensis* E+U extract were determined by the method of Decker [32], with minor modifications. An ethanol solution of the samples (1 mL) at various concentrations (0.05, 0.1, 0.2, 0.4, 0.8, 1.0 and 1.2 mg/mL) was mixed with methanol (3.7 mL), FeCl_2_∙4H_2_O (2 mM, 0.1 mL) and ferrozine (5 mM, 0.2 mL). The mixture stood in the dark for 10 min. The control contained all the reagents without the sample and was used as a blank. The metal chelating activity was determined by measuring the absorbance at 562 nm using a spectrophotometer. The metal chelating activity of EDTA was also determined for comparison. The metal chelating activity was calculated as (1 − absorbance of sample/absorbance of control) × 100%.

#### 3.6.3. Total Antioxidant Activity

The total antioxidant activities of all the samples of *S. sinensis* E+U extract were determined by the method of Re *et al.* [33], with minor modifications. An ABTS radical cation (ABTS^+^) solution was prepared through the reaction of ABTS (7 mM) with potassium phosphate (2.45 mM), following incubation at room temperature in the dark for 12 h. The ABTS^+^ solution was then diluted with 95% ethanol to obtain an absorbance of 0.70 ± 0.02 at 734 nm. Each sample (2 mL) or Trolox standard (2 mL) was added to ABTS^+^ solution (2 mL) and mixed vigorously. The reaction mixture was allowed to stand at room temperature for 6 min, and the total antioxidant activity was determined by measuring the absorbance at 734 nm using a spectrophotometer. The control contained all the reagents without the sample and was used as a blank. The total antioxidant activity of vitamin C was also determined for comparison. The total antioxidant activity was calculated as (1 − absorbance of sample/absorbance of control) × 100%.

#### 3.6.4. Assay of Superoxide Radical Scavenging Capacity

The superoxide radical scavenging capacity was determined according to the method of Jing and Zhao [34]. Mixture solutions contained Tris-HCl buffer (50 mM, pH 8.2, 4.5 mL), 25 mM pyrogallol solution (0.4 mL) and sample (1 mL) incubated at 25 °C for 5 min. Then, 8 mM HCl solution (1 mL) was dripped into the mixture promptly to terminate the reaction. The absorbance was measured at 420 nm. Vitamin C was used as the positive control. The superoxide radical scavenging capacity was calculated by the following formula:

Scavenging capacity (%) = [1 − (A_1_ − A_2_)/A_0_] × 100% (1)

where A_0_ is the absorbance of the control, A_1_ is the absorbance of the sample and A_2_ is the absorbance of the sample only (Tris-HCl buffer instead of pyrogallol solution).

### 3.7. Mushroom Tyrosinase Inhibitor Assay

The mushroom tyrosinase activity was determined according to the method of Rahman [35]. The tyrosinase activity was determined using L-DOPA as a substrate. Briefly, 700 units/mL tyrosine solution (50 μL) was dissolved in 0.1 M of phosphate buffer (pH 6.8), then 50 μL of each of the different concentrations of the *S. sinensis* E+U extract solution was added to each well of a 96-well plate and mixed. The assay mixture was pre-incubated at room temperature for 10 min; 2.5 mM L-DOPA in 0.1 M of phosphate buffer (pH 6.8, 100 μL) was then added to each well and incubation was continued for 20 min at room temperature. The amount of dopachrome formed in the reaction mixture was determined against the blank (solution without enzyme) at 475 nm in a microplate reader (Multiskan GO, Thermo Scientific, Waltham, MA). Kojic acid was used as a standard tyrosinase inhibitor in order to confirm that the assay was working. The percentage of tyrosinase activity was calculated as follows:

Tyrosinase activity (%) = [(A − B)/(C − D)] × 100 (2)

where A is the absorbance of the reaction mixture containing the test sample and mushroom tyrosinase, B is the absorbance of the blank sample containing the test sample but without mushroom tyrosinase, C is the absorbance of the reaction mixture without the test sample and with mushroom tyrosinase and D is the absorbance of the well without either the test sample or mushroom tyrosinase (L-DOPA alone). 

### 3.8. Statistical Analysis

Results are expressed as mean ± SD. Differences among the groups were subjected to a one-way ANOVA (analysis of variance) followed by Duncan’s multiple range. Statistical significance was accepted when a *p*-value was less than 0.05. 

## 4. Conclusions

The total flavonoid compound and FA contents of extracts from *S. sinensis* prepared by five methods were investigated in the present study. E+U was determined to be a better extraction technique for obtaining higher amounts of flavonoid compounds and FA contents than the other four methods. Furthermore, flavonoid compounds and FA from *S. sinensis* showed excellent antioxidant activity in multiple test systems and was a good inhibitor of tyrosinase activity. Based on the results obtained in this study, flavonoid compounds and FA from *S. sinensis* could be beneficial to the antioxidant protection system and inhibit tyrosinase activity in health foods and cosmetic products, which would protect the human body against oxidative damage, both internally and externally. 
